# Electronic smoking devices. We cannot let our young people go down this path of addiction and disease

**DOI:** 10.36416/1806-3756/e20250121

**Published:** 2025-06-20

**Authors:** Luiz Fernando Ferreira Pereira, Maria Enedina Claudino Aquino Scuarcialupi, Maria Vera Cruz de Oliveira Castellano, Carlos Leonardo Carvalho Pessôa, Ramiro Dourado, Comissão de tabagismo da SBPT 2025-26

**Affiliations:** 1. Hospital das Clínicas, Universidade Federal de Minas Gerais - UFMG - Belo Horizonte (MG) Brasil.; 2. Cancer Center, Oncoclínas e Hospital Biocor, Rede D’Or, Belo Horizonte (MG) Brasil.; 3. Afya Faculdade de Ciências Médicas da Paraíba - AFYA-Paraíba - Cabedello (PB) Brasil.; 4. Coordenação de Doenças Respiratórias, Centro de Referência Multiprofissional de Doenças Raras, Hospital Universitário Lauro Wanderley, Faculdade de Medicina, Universidade Federal da Paraíba -UFPB -- João Pessoa (PB) Brasil.; 5. Hospital do Servidor Público Estadual de São Paulo - IAMSPE-- São Paulo (SP) Brasil.; 6. Disciplina de Pneumologia, Faculdade de Medicina, Universidade Federal Fluminense - UFF - Niterói (RJ) Brasil.; 7. Centro Universitário do Planalto Central Aparecido Santos - Uniceplac - Brasília (DF) Brasil.; 8. Hospital das Clínicas, Universidade Federal de Goiânia, Goiânia (GO) Brasil.; 9. Hospital Regional do Gama, Gama (DF) Brasil.

Worldwide, there are more than 1.25 billion smokers, of whom 80% live in low-income countries, and the majority want to quit smoking. In recent decades, the tobacco industry has invested in new forms of smokeless nicotine consumption, notably moist sachet and snus and, especially, electronic smoking devices (ESDs), also known as electronic cigarettes, vapes, pods, and e-cigs.

Public policies to control smoking in Brazil, including the free treatment program coordinated by the National Ministry of Health and the National Cancer Institute, have made the country a model of reducing cigarette smoking, which fell from more than 34% in the 1980s to less than 10% in 2023.^1^

In contrast to cigarette smoking, which has shown a slow, progressive reduction in several regions of the world, the use of ESDs is growing at an alarming rate, partly due to curiosity, technological appeal, ease of acquisition, and massive dissemination on social media. In some countries, more than 25% of young people have used an ESD in the last 30 days.^2^ In Brazil, there are an estimated 3 million ESD users, with a recent increase in current (daily or occasional) use among people 18-24 years of age, which reached 6.6% in 2023.^2^ ^-^ ^4^

In the media, especially on social media, false or exaggerated statements are often spread, some of which distort the conclusions of scientific articles, in favor of vapes, such as the following: they only release water vapor; they release less nicotine than do cigarettes; they do not pollute the environment; they have fewer harmful health effects in comparison with cigarettes; and they help people quit smoking.

In recent years, the tobacco industry has vehemently argued that authorizing the manufacture and sale of standardized, certified ESDs, with a maximum of 20 mg/ml of nicotine, would guarantee the use of quality products, reduce black-market activity, and increase tax revenues from the sale of tobacco derivatives.

All of these arguments in favor of ESD use are fallacious.

Validated studies with strong scientific evidence, as well as national and international recommendations, including those of the World Health Organization, prove exactly the opposite.^5^ ^-^ ^8^ The main arguments against the use of ESD are as follows:

ESDs not only release water vapor; their white, odorless vapor contains propylene glycol, glycerol, metals, particulates, carcinogens, and a growing number of substances, many with potential health risks.^9^

In addition to making it easier to inhale the vapor, the latest generation of ESDs are less irritating to the throat, release a greater quantity of particulates and nicotine (salts that are better absorbed by the lungs) than do regular cigarettes, and can be augmented with more than 16,000 flavorings, which incentivize the initiation and maintenance of their use, especially among younger people.^8^ ^,^ ^10^

The use of vapes causes nicotine dependence and other health problems such as coughing, dyspnea, oral/dental changes, cardiovascular risks, risk of cancer, respiratory diseases, and intense withdrawal symptoms after reducing or stopping use, due to the high daily consumption of high concentrations of nicotine.^5^ ^,^ ^6^ ^,^ ^8^ In addition, vaping can cause health problems due to battery explosions or intoxication due to ingestion of liquid from the reservoirs.

ESDs can also cause a febrile lung disease, associated with gastrointestinal manifestations with a high risk of severity and death, known as e-cigarette or vaping product use-associated lung injury (EVALI), especially when loaded with marijuana derivatives, vitamin E, or both.^11^

In Brazil, one out of every three regular cigarettes consumed is obtained from the black market.^12^ Tax revenue from the manufacture and sale of tobacco generates less than one tenth of the amount the country spends on treating diseases, pays for early retirements, and loses from deaths due to smoking.^13^ Regulating vapes will not solve the problem of the black market, which would continue to sell ESD with a high concentration of nicotine and might increase the consumption of certified products (Table 1).

Although the illegal sales of regular cigarettes and vapes remain high in Brazil, governmental agencies charged with health oversight, consumer protection, and tax auditing have ramped up their activities, which include inspections, educational actions, fines, and seizures of property.

A young person who starts vaping triples their risk of starting to smoke regular cigarettes and increases their risk of starting to use other drugs.^14^

Smoking cessation treatment is effective and is based on behavioral support combined with nicotine replacement therapy (patches, gum, lozenges, or any combination of those), varenicline (temporarily unavailable in Brazil), or bupropion. Although controversy persists regarding the role of ESDs in smoking cessation, a review of the literature demonstrated that they are superior to nicotine patches, although with a success rate much lower than that obtained in structured programs.^15^ However, that small gain is offset by the fact that more than half of individuals who quit smoking do not stop vaping, remaining dependent on nicotine, and many revert to smoking regular cigarettes alone.^16^ ^,^ ^17^ To make matters worse, many patients who do not quit smoking continue to use ESDs, becoming dual users, with increased risks to their health.

**Table 1:** Practical comparisons between regular cigarettes and e-cigarettes.

Aspect	Regular cigarettes	E-cigarettes^a^ ESDs
Black market	33%^12^	100%
Released substances	7,500 (250 that are harmful to health)	From tens to hundreds^b^ (Dozens that are harmful to health)
Nicotine concentration	1 regular cigarette: ≤ 1 mg	1 ESD capsule ≤ 50 mg/mL^c^
Amount of nicotine consumed	1 pack/day or 600 cigarettes/month 15 puffs/cigarette or 300 puffs/day 20 cigarettes/day = 20 mg 600 mg of nicotine/month	1 ESD 13 mL, 50 mg/mL, 10,000 puffs/unit Tens to hundreds of puffs/day 333 puffs/day = 21.6 mg 650 mg of nicotine/month

ESDs: electronic smoking devices. ^a^ Many generations and hundreds of nonstandardized brands make it difficult to assess the substances that are released and the harmful effects. 4th-generation ESDs release more particulates, metals, and nicotine than do regular cigarettes. The nicotine salts in 4th-generation cartridges (pods) are better absorbed by the lungs, and > 16,000 flavorings are available. ^b^ Teharini et al.^9^ detected > 2,000 substances. ^c^ Elfbar, one of the most widely sold disposable ESDs in several countries, has a reservoir of up to 50 mL, releases ≤ 50 mg of nicotine/mL and ≤ 40,000 puffs - www.wolfshopbrasil.com

In 2024, the *Agência Nacional de Vigilância Sanitária* (ANVISA, Brazilian Health Regulatory Agency), after extensive review and discussion, ratified Collegiate Board Resolution no. 855 (4/23/2024), which imposed a nationwide ban on the manufacture, import, storage, distribution, marketing, and advertising of vapes, as well as on their use in closed public spaces.^18^

Historically, the greatest concern of the tobacco industry has never been the health of its users; quite the opposite, its main objective has always been to create ever more nicotine addicts, who will, as we know, use its products for decades.

For the aforementioned reasons, the Brazilian Thoracic Association, together with other national and international associations, ratifies its position against allowing the manufacture, commercialization, and promotion of vapes, as well as vaping in enclosed spaces, and believes that a massive educational campaign, aimed at young people, is essential and urgent, to try to reverse the growing use of these devices in Brazil.^19^ ^,^ ^20^

The use of ESDs is not a step forward; it is a step backward in the ongoing quest for a tobacco- and nicotine-free world.
